# Supplementary material for: Tumor stiffening reversion through collagen crosslinking inhibition improves T cell migration and anti-PD-1 treatment
Source: eLife. 2021 Jun 9;10:e58688. doi: 10.7554/eLife.58688 (PMC8203293; doi:10.7554/eLife.58688)
Supplement: Supplementary file 1. [file elife-58688-supp1.pptx]

## Slide 1
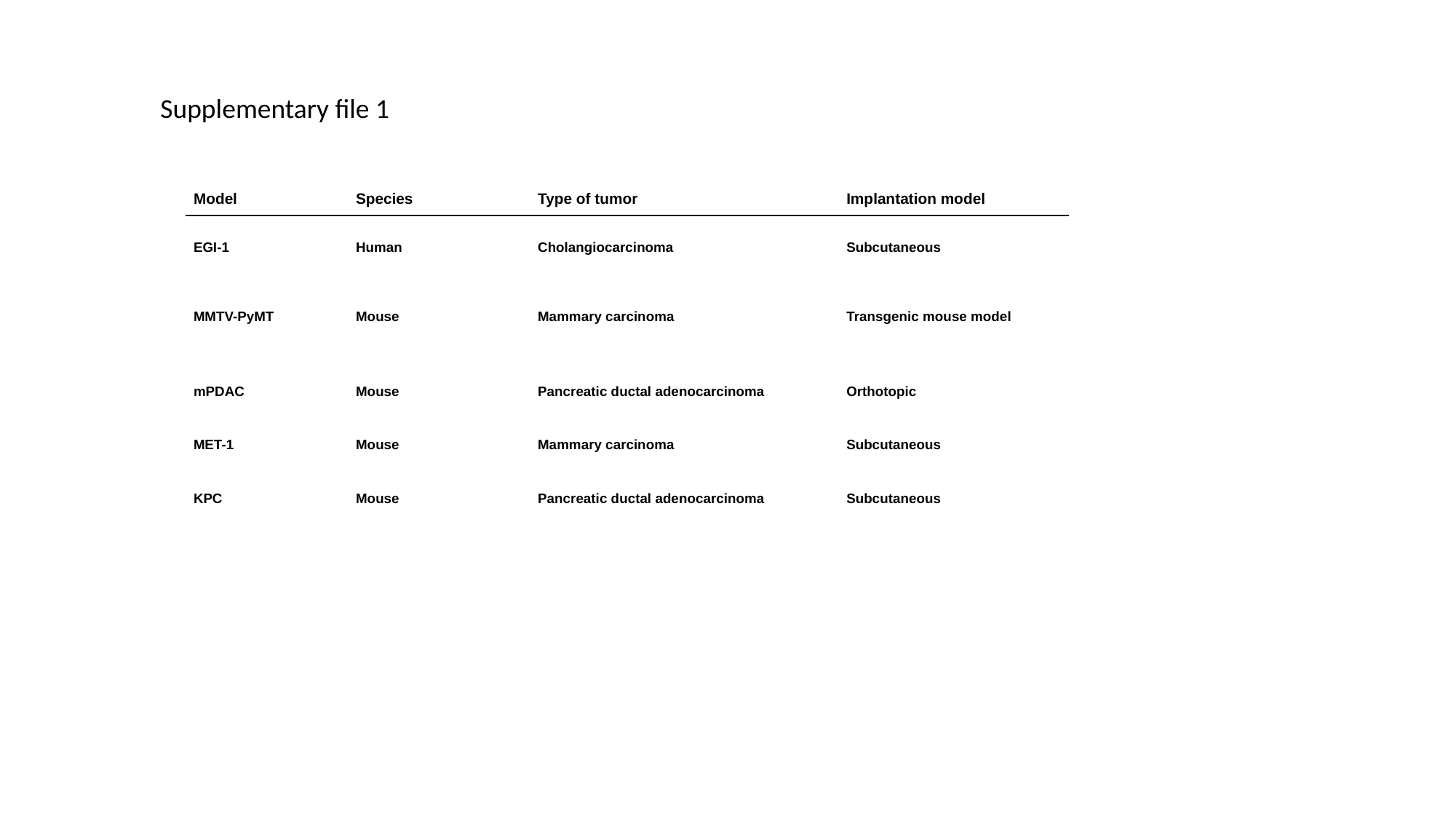

Supplementary file 1
| Model | Species | Type of tumor | Implantation model |
| --- | --- | --- | --- |
| EGI-1 | Human | Cholangiocarcinoma | Subcutaneous |
| MMTV-PyMT | Mouse | Mammary carcinoma | Transgenic mouse model |
| mPDAC | Mouse | Pancreatic ductal adenocarcinoma | Orthotopic |
| MET-1 | Mouse | Mammary carcinoma | Subcutaneous |
| KPC | Mouse | Pancreatic ductal adenocarcinoma | Subcutaneous |
