# Supplementary material for: Tumor stiffening reversion through collagen crosslinking inhibition improves T cell migration and anti-PD-1 treatment
Source: eLife. 2021 Jun 9;10:e58688. doi: 10.7554/eLife.58688 (PMC8203293; doi:10.7554/eLife.58688)
Supplement: Supplementary file 2. — Mean stiffness of tumors with volume > 600 mm3 measured with SWE. Percentage of stiff regions of tumors with volume > 600 mm3. Tumor architecture was characterized by the percentage of the tumor covered by the stromal compartment, estimated from HES images; collagen fiber width and length, calculated from SHG images; percentage of red-orange birefringent fibers combining Red Sirius staining and polarized microscopy, orange-red fibers correspond to thick and packed regions. EGI-1 (n = 30 mice/group from three independent experiments); KPC (n = 34 mice/group from three independent experiments); MMTV-PyMT (n = 5 mice/group, 10 tumors per mouse from two independent experiments); mPDAC (n = 7 mice/group from two independent experiments); and MET-1 (n = 12 mice/group from two independent experiments). [file elife-58688-supp2.pptx]

## Slide 1
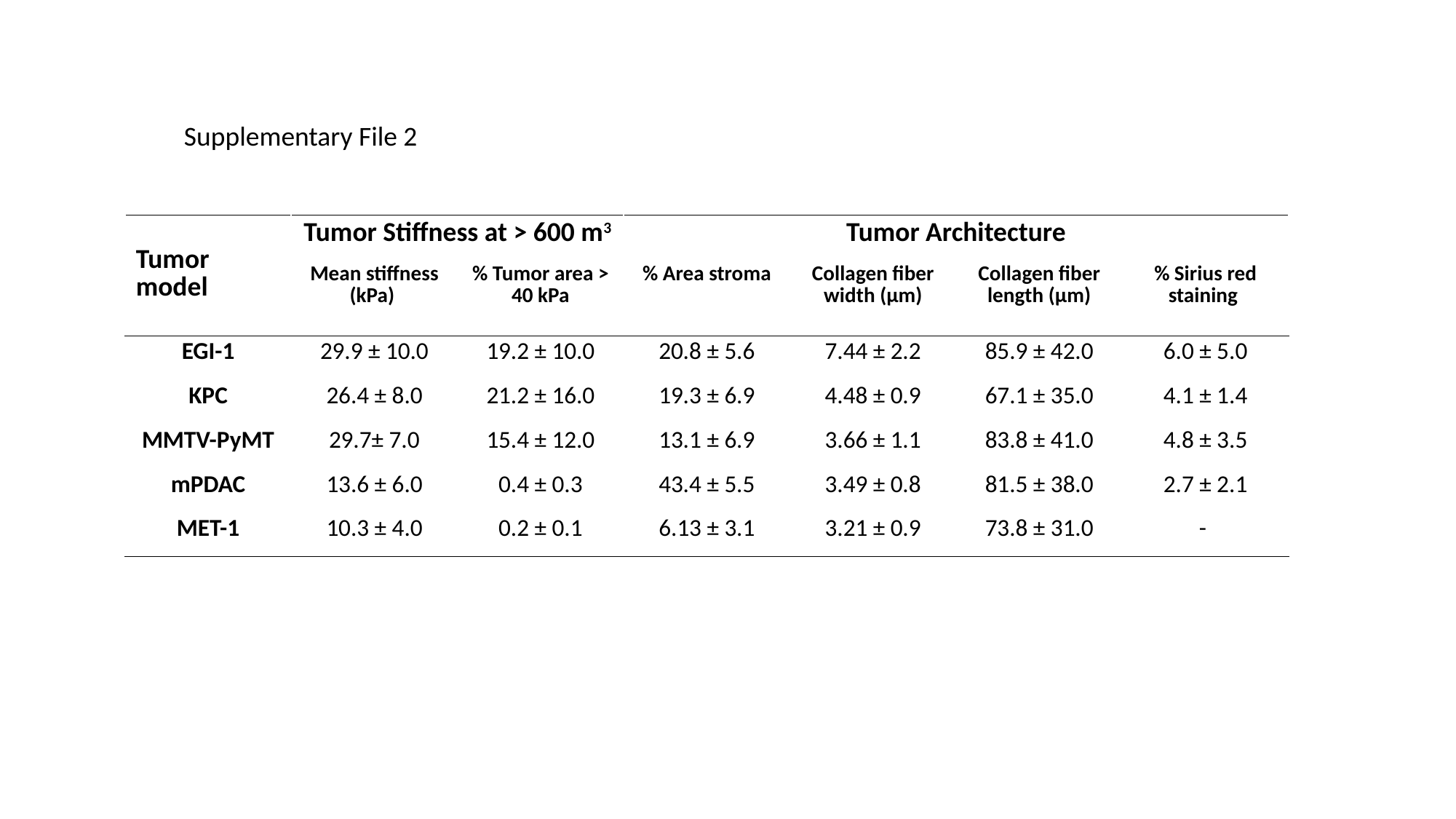

Supplementary File 2
| Tumor model | Tumor Stiffness at > 600 m3 | | Tumor Architecture | | | |
| --- | --- | --- | --- | --- | --- | --- |
| | Mean stiffness (kPa) | % Tumor area > 40 kPa | % Area stroma | Collagen fiber width (µm) | Collagen fiber length (µm) | % Sirius red staining |
| EGI-1 | 29.9 ± 10.0 | 19.2 ± 10.0 | 20.8 ± 5.6 | 7.44 ± 2.2 | 85.9 ± 42.0 | 6.0 ± 5.0 |
| KPC | 26.4 ± 8.0 | 21.2 ± 16.0 | 19.3 ± 6.9 | 4.48 ± 0.9 | 67.1 ± 35.0 | 4.1 ± 1.4 |
| MMTV-PyMT | 29.7± 7.0 | 15.4 ± 12.0 | 13.1 ± 6.9 | 3.66 ± 1.1 | 83.8 ± 41.0 | 4.8 ± 3.5 |
| mPDAC | 13.6 ± 6.0 | 0.4 ± 0.3 | 43.4 ± 5.5 | 3.49 ± 0.8 | 81.5 ± 38.0 | 2.7 ± 2.1 |
| MET-1 | 10.3 ± 4.0 | 0.2 ± 0.1 | 6.13 ± 3.1 | 3.21 ± 0.9 | 73.8 ± 31.0 | - |
