# Supplementary material for: Tumor stiffening reversion through collagen crosslinking inhibition improves T cell migration and anti-PD-1 treatment
Source: eLife. 2021 Jun 9;10:e58688. doi: 10.7554/eLife.58688 (PMC8203293; doi:10.7554/eLife.58688)
Supplement: Supplementary file 3. — Results are shown as mean ± SD. [file elife-58688-supp3.pptx]

## Slide 1
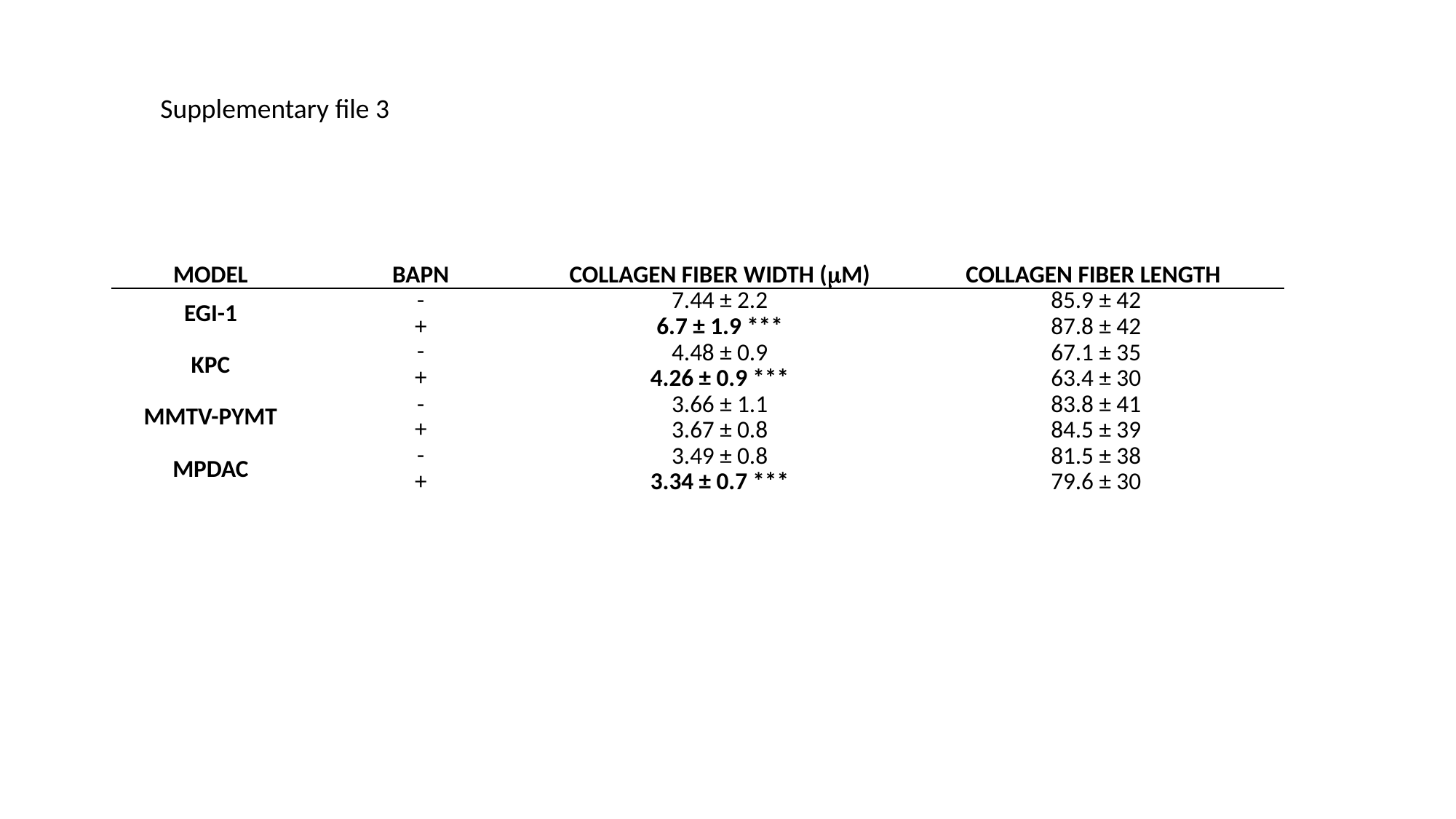

Supplementary file 3
| Model | BAPN | Collagen fiber width (μm) | COLLAGEN FIBER LENGTH |
| --- | --- | --- | --- |
| EGI-1 | - | 7.44 ± 2.2 | 85.9 ± 42 |
| | + | 6.7 ± 1.9 \*\*\* | 87.8 ± 42 |
| KPC | - | 4.48 ± 0.9 | 67.1 ± 35 |
| | + | 4.26 ± 0.9 \*\*\* | 63.4 ± 30 |
| MMTV-PyMT | - | 3.66 ± 1.1 | 83.8 ± 41 |
| | + | 3.67 ± 0.8 | 84.5 ± 39 |
| mPDAC | - | 3.49 ± 0.8 | 81.5 ± 38 |
| | + | 3.34 ± 0.7 \*\*\* | 79.6 ± 30 |
