# Supplementary material for: Tumor stiffening reversion through collagen crosslinking inhibition improves T cell migration and anti-PD-1 treatment
Source: eLife. 2021 Jun 9;10:e58688. doi: 10.7554/eLife.58688 (PMC8203293; doi:10.7554/eLife.58688)
Supplement: Supplementary file 4. — Migration of activated PBT overlaid onto fresh tumor slices was analyzed in EGI-1 and MMTV-PyMT model, whilst resident tumor infiltrated T lymphocyte migration was analyzed in mPDAC and KPC models (* p-value<0.05, ***p-value<0.001, Students’ t-test, n = 3–12 mice/group from three experiments, 70–250 T cells per slice analyzed). T cell migration was analyzed at the endpoint of the experiment: day 30 for EGI-1, day 40 for KPC, day 55 for MMTV-PyMT and day 21 for mPDAC. T cell infiltration (CD8/mm2) calculated from immunofluorescence images. Results are shown as mean ± SD. Illustrative images of T cell migration tracks in EGI-1 tumor model. Tumor stroma (fibronectin) in red, tumor cells (EpCAM) in blue and T cells (Calcein) in green. Tracks are color-coded to illustrate track displacement. Scale bar = 100 µm. See also Videos 1 and 2. TILs: tumor-infiltrating T lymphocytes. [file elife-58688-supp4.pptx]

## Slide 1
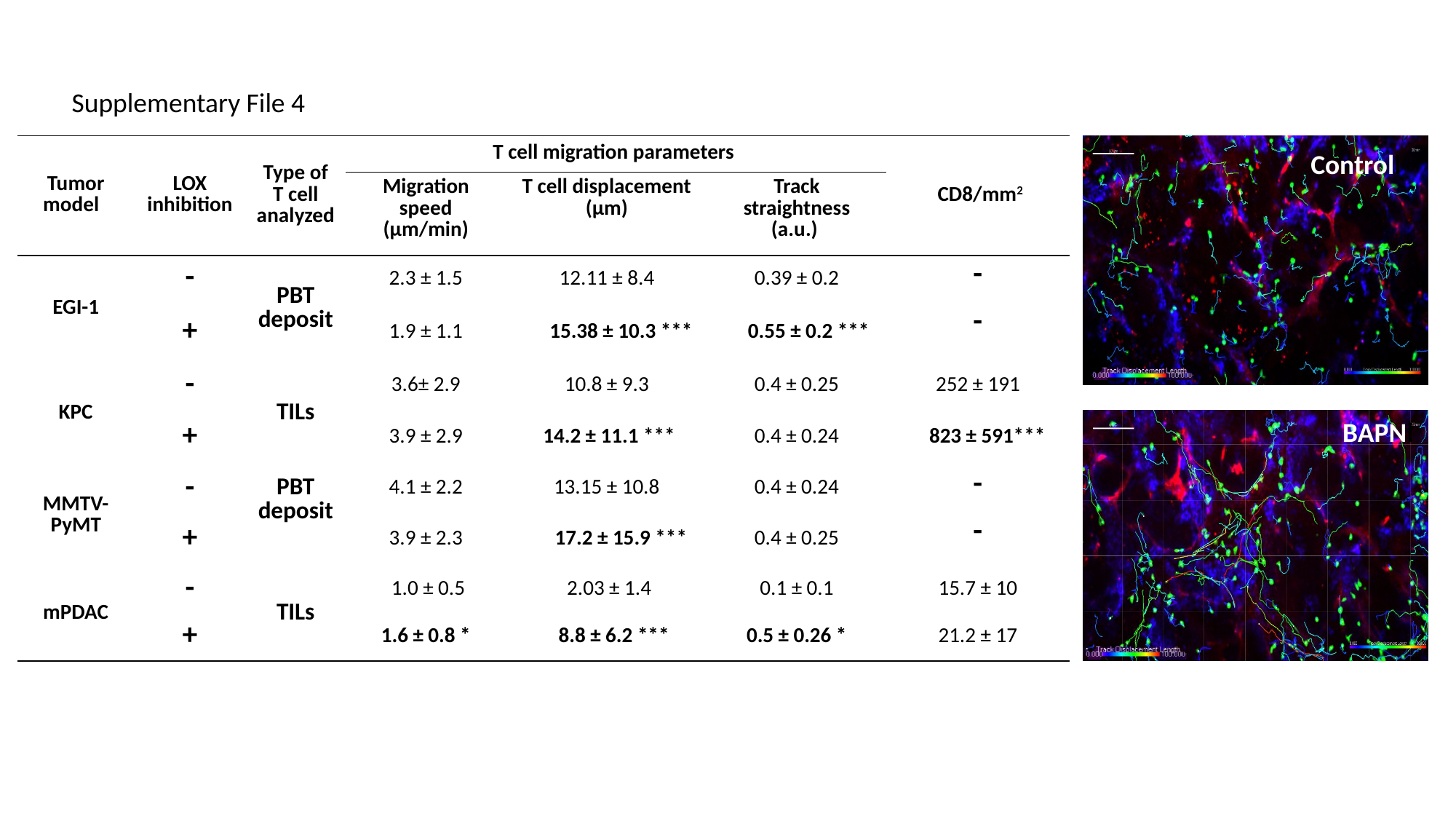

Supplementary File 4
| Tumor model | LOX inhibition | Type of T cell analyzed | T cell migration parameters | | | CD8/mm2 |
| --- | --- | --- | --- | --- | --- | --- |
| | | | Migration speed (µm/min) | T cell displacement (µm) | Track straightness (a.u.) | |
| EGI-1 | - | PBT deposit | 2.3 ± 1.5 | 12.11 ± 8.4 | 0.39 ± 0.2 | - |
| | + | | 1.9 ± 1.1 | 15.38 ± 10.3 \*\*\* | 0.55 ± 0.2 \*\*\* | - |
| KPC | - | TILs | 3.6± 2.9 | 10.8 ± 9.3 | 0.4 ± 0.25 | 252 ± 191 |
| | + | | 3.9 ± 2.9 | 14.2 ± 11.1 \*\*\* | 0.4 ± 0.24 | 823 ± 591\*\*\* |
| MMTV-PyMT | - | PBT deposit | 4.1 ± 2.2 | 13.15 ± 10.8 | 0.4 ± 0.24 | - |
| | + | | 3.9 ± 2.3 | 17.2 ± 15.9 \*\*\* | 0.4 ± 0.25 | - |
| mPDAC | - | TILs | 1.0 ± 0.5 | 2.03 ± 1.4 | 0.1 ± 0.1 | 15.7 ± 10 |
| | + | | 1.6 ± 0.8 \* | 8.8 ± 6.2 \*\*\* | 0.5 ± 0.26 \* | 21.2 ± 17 |
Controll
BAPNl
